# Diagnostic power of one-step and two-step RT-qPCR methods to SARS‑CoV‑2 detection

**DOI:** 10.1186/s12879-022-07478-0

**Published:** 2022-05-31

**Authors:** Asra Malekshahi, Sayyad Khanizadeh, Shirzad Fallahi, Gholamreza Talei, Mehdi Birjandi, Faezeh Hajizadeh

**Affiliations:** 1 Department of Virology, School of Medicine, Lorestan University of Medical Sciences, Khorramabad, Iran; 2 Hepatitis Research Center, Lorestan University of Medical Sciences, Khorramabad, Iran; 3 Department of Parasitology and Mycology, School of Medicine, Lorestan University of Medical Sciences, Khorramabad, Iran; 4 Department of Biostatistics and Epidemiology, School of Health and Nutrition, Lorestan University of Medical Sciences, Khorramabad, Iran

**Keywords:** SARS-COV-2, Real-Time PCR, COVID-19, Curve, Roc

## Abstract

**Background:**

Coronavirus-2019 (COVID-2019) is a novel coronavirus known as Acute Respiratory Syndrome (SARS-CoV-2). The premier standard test for SARS-CoV-2 diagnosis is a one-step RT-qPCR method, which requires specific probes and reagents. Therefore, detection on a large scale is expensive and cannot be very accurate.

**Methods:**

A cost-effective technique based on SYBR green was evaluated in the current study. The specific primers for S and N genes were designed, then performed the cross-reactivity test with other coronavirus and respiratory viruses positive samples. Moreover, the analytical sensitivity test was carried out with 8 dilutions (1:10). Lastly, the SARS-CoV-2 clinical samples (n = 210) were tested by these two methods, and receiver operating characteristic (ROC) analysis was performed to investigate the incremental diagnostic value of each gene in the study methods.

**Results:**

The two-step method detected up to 6th dilutions of the SARS-CoV-2 samples and did not show any amplification of the positive samples of other respiratory viruses. ROC analysis revealed a diagnostic ability of the two-step method for SARS-CoV-2 with an area under the ROC curve of ≥ 0.7 (P ˂ 0.05) and relatively high sensitivity and specificity. The combination of N and S genes increased the sensitivity up to 88%, specificity up to 86%, and area under the ROC curve up to 0.85 (95% confidence interval (95% CI) 0.72 to 0.93, P = 0.0461).

**Conclusion:**

Our findings indicated that the two-step method has comparable sensitivity and specificity to the one-step method. Therefore, this method can be considered a potential diagnostic method for diagnosing and monitoring COVID-19 patients. It suggests that when the one-step RT-qPCR method is not available, the two-step RT-qPCR can be used to identify SARS-CoV-2.

## Introduction

Coronavirus disease-2019 (COVID-2019) is known as Severe Acute Respiratory Syndrome Coronavirus 2 (SARS-CoV-2). The COVID-19 outbreak by the new coronavirus strain was recognized as a pandemic by the World Health Organization (WHO) on 11 March 2020 [1]. COVID-19 has high infections and mortality worldwide; moreover, there have been other consequences, including damage to education, social interactions, and the world economy [2, 3]. Isolation, social distancing, and early detection have been the main tools in the fight to interrupt the chain of viral transmission. Some individuals may be asymptomatic, making it difficult to diagnose the virus [4]. The SARS-CoV-2 testing capacity is an important issue worldwide. Development of the testing capacity is critical for quick detection and screening of cases during outbreaks, particularly in developing countries where the availability of supplies or infrastructure to carry out the real-time PCR test is limited [5]. Consequently, to develop alternative methods for SARS-CoV-2 detection that might be faster or cheaper to perform, different research has been conducted, such as sample pooling of RNA extracts [6], two steps endpoint RT‑PCR with agarose gel electrophoresis [7], loop-mediated isothermal amplification (LAMP) [8, 9], multiplex PCR [10], droplet digital PCR (ddPCR) [11] or even protocols based on CRISPR–Cas12 [12]. Reverse transcription-quantitative real-time PCR (RT-qPCR) is the foremost test for diagnosing SARS-CoV-2 in a clinic [13]. The RT-qPCR is working based on the amplification of the RNA target followed by incremental fluorescence dye. It is performed at two levels, i.e., TaqMan probe-based and SYBR Green-based RT-qPCR. However, each has some advantages and disadvantages. Although both methods utilize expensive reagents and equipment [14, 15], the TaqMan probe-based method is more costly because of using a specific probe.

On the other hand, the SYBR Green-based method has a lower accuracy as primer tends to form non-specific products and primer-dimer, although trained technicians can avoid it. In addition, this method has been proposed and used for testing different types of viruses [13, 16, 17]. Altogether, the one-step method is preferred to the two-step manner [15, 18]. The present study compared the one-step TaqMan probe-based method and the two-step SYBR Green-based method.

## Material and method

### Samples recruitment

Positive (n = 110) and negative (n = 100) specimens were recruited from the COVID-19 Reference Laboratory, Lorestan University of Medical Sciences Khorramabad, Iran; based on Taq Man RT-qPCR results recommended by WHO [19]. All positive subjects have a cycle threshold (Ct) value < 40, and negative samples have a Ct value > 40. Swab samples were collected in the safety level 2 laboratory. Our study was performed between June 2019 and November 2021, and the Committee approved the investigation of Clinical Research Ethicals in Lorestan University of Medical Sciences, Khorramabad, Iran (Ethical code: IR.LUMS.REC.1400.115). Demographic characteristics were recorded at the time of sampling.

### Sample preparation

The swab was inserted into the nasopharyngeal and oropharyngeal ducts of individuals who had COVID-19 symptoms and put the swabs in 2 ml of fresh viral transport media (VTM). The samples were transferred into the refrigerator for RT-qPCR assessment and stored at 4 °C.

### Pooled sample and serial dilution

We used the pooling sample process to enhance collected samples’ analytical capacity and efficiency of collected samples. The 20 positive specimens were collected into a container and diluted in the ratio of 1:10 (8-times). Afterward, the copy number of each concentration was calculated at Pishtaztab Lab using by SARS-CoV-2 standard sample.

### Viral genome extraction and RT-qPCR

According to the manufacturer’s protocol, total RNA was extracted by RNJia Virus Kit solutions (Rojeh Technology Company, Tehran, Iran). The total amount of extracted RNA was evaluated using a nanodrop 1000 spectrophotometer (Wilmington, USA) and qualified by agarose gel electrophoresis. Reverse transcription of extracted RNA into cDNA in a two-step manner was applied using a cDNA synthesis kit (Takara, Japan). Quantitative reverse transcription PCR (RT-qPCR) was implemented using SYBR green (Prime Script RT Master Mix, Takara) and TaqMan probe (Pishtaztab, Tehran, Iran), and MIC PCR (BioMolecular System, Australia). For SYBR Green-based RT-qPCR method, Master Mix Green SYBR Green and specific primers designed for S and N genes were used. For this method, each 20 μl reaction contained 10 μl of Master Mix, 0.6 μl of primer (0.25 μM final concentration each), 7.8 μl of nuclease-free water, and 1 μl of cDNA with the following conditions: pre-denaturation at 95 °C for 35 s, followed by 40 cycles of denaturation at 95 °C for 5 s, and extension at 62 °C for 1 min. The reverse and forward primer sequences for the two-step method (SYBR Green-based RT-qPCR method) (Table 1) were then used.

**Table 1:** Sequence of primers designed for two step RT-qPCR method

Gene	Primer	Primer length (bp)	Product length
S	F 5ʹ-TCCATCAAAACCAAGCAAGA-3ʹ	20	245 bp
	R 5ʹ-CACCAAAGGTCCAACCAGAA-3ʹ	20	NC_045512v2:23,986 + 24,230
N	F 5ʹ-CTGGACTTCCCTATGGTGCT-3ʹ	20	284 bp
	R 5ʹ-ATTGCCAGCCATTCTAGCAG-3ʹ	20	NC_045512v2:28,629 + 28,912

The TaqMan probe-based method was performed using a commercial one-step RTqPCR Kit (Pishtaztab, Tehran, Iran). This kit’s probe and primer mixtures were designed using the dual-target gene method, which simultaneously identifies protected genomic sequences of the RdRp region and the N-protein nucleocapsid. The one-step qPCR was performed according to the manufacturer’s instructions. Briefly, each 20 μl reaction contained 9 μl of Master Mix (Enzyme mix + 5× buffer real-time PCR), 1 μl of primer, probe (0.3 μM final concentration each), 5 μl of nuclease-free water, and 5 μl of RNA. Thermal cycling was: 50 °C for 20 min for reverse transcription, inactivation of reverse transcriptase at 95 °C for 3 min, and then 40 cycles of 94 °C for 10 s and 55 °C for the 40 s.

Non-template control (nuclease-free water) was included in every qPCR run as a negative control.

### Determination of analytical specificity

To confirm the analytical specificity of the SYBR Green real-time RT-PCR, the RNA was extracted from OC43, 229E, NL63, Influenza A virus (H1N1), human rhinovirus, and human respiratory syncytial virus samples. After the cDNA synthesis, the two-step manner PCR used the SARS-CoV-2 primers was carried out on the samples.

### Static analysis

The crud data was analyzed statically by SPSS (19.03) and MedCalc (14.8.1) software. The ROC curve analysis was applied to find the sensitivity and specificity of each two methods. Quantitative and qualitative variables were presented as mean ± SD and frequency (percent), respectively. A P-value < 0.05 was considered statistically significant.

## Results

### Subjects and specimens

**Fig. 1:**
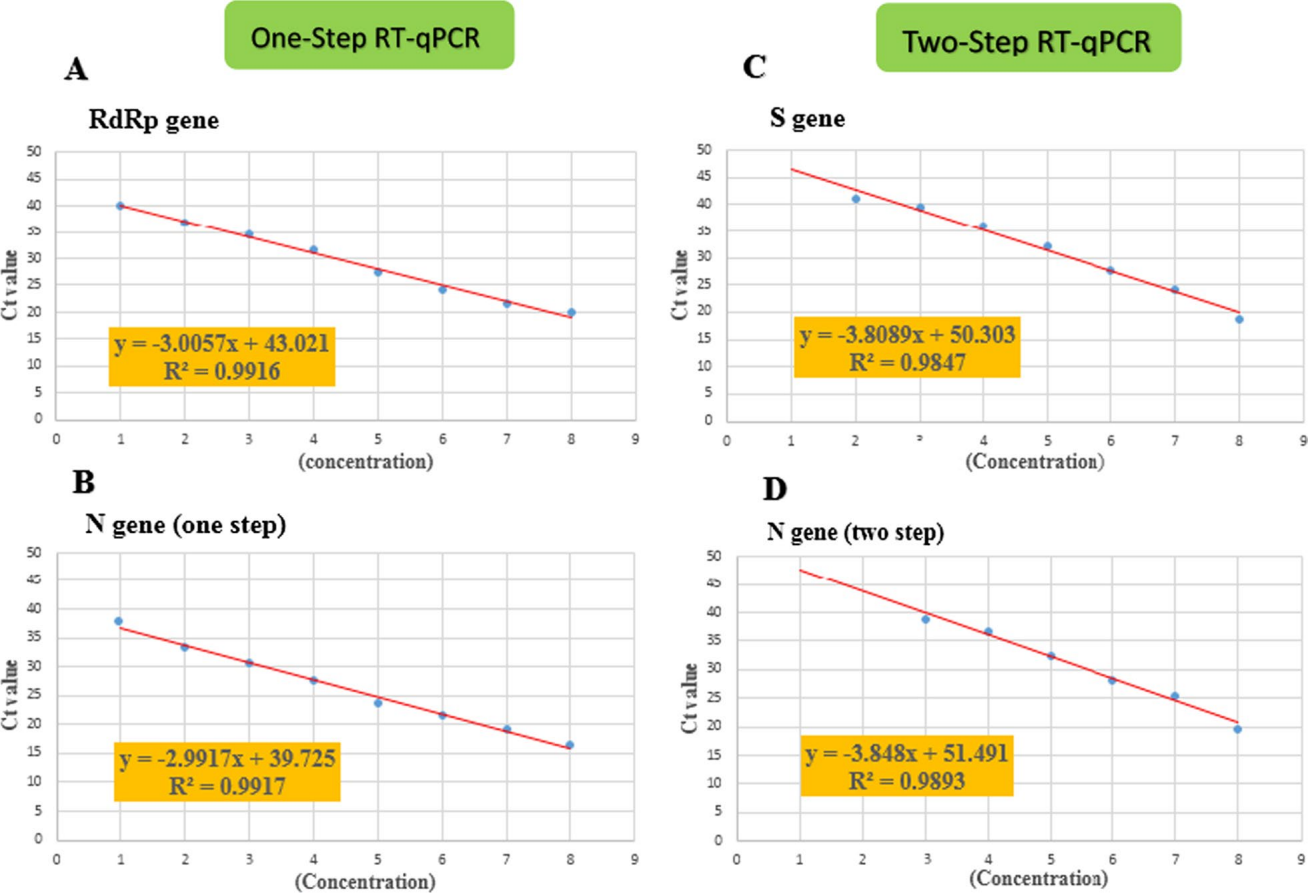
The Calibration curves of one-step and two-step RT-qPCR. Serially diluted RNA containing RdRp (**A**), N (**B**, **D**), S (**C**) targets were amplified and analyzed in one-step (**A**, **B**) and two-step (**B**, **D**) RT-qPCR protocols. The threshold cycle (Ct) values were plotted against the concentration of RNA standards ng/µl. The coefficient of determination (R2) and the linear regression curve (y) for everyone was calculated

A total of 210 swap samples into VTM, including 110 positives and 100 negatives, were recruited in the current investigation. We matched the study groups regarding age, gender, location, lung disease, and clinical features, so there were no statistically remarkable differences (Table 2). Afterward, the positive samples were pooled together and diluted for 8-times, and finally, the genome COVID-19 copy numbers and Ct values for each concentration were calculated (Table 3). Furthermore, the standard curves for S and N genes in two-step and RdRp and N genes in one-step RT-qPCR have been shown in Fig. 1.

**Table 2:** Demographic characteristics of the samples

Data	Finding	Total n = 210	Positive sample n = 110	Negative sample n = 100	P value
		*n* (%)	*n* (%)	*n* (%)	
Gender	Male	52.00%	53.50%	50.40%	0.083
	Female	48.00%	46.50%	49.60%	
Age (years)	11–40	60.50%	59.80%	61.30%	
	41–70	35.50%	36.20%	34.70%	0.076
	70<	3.90%	3.90%	3.90%	
Location	City	53.90%	70.10%	37.80%	–
	Village	46.10%	29.90%	62.20%	
Lung disease	Yes	1.60%	3.10%	0.00%	–
	No	98.40%	96.90%	100.00%	
Fever	Yes	9.10%	9.40%	8.70%	0.158
	No	90.90%	90.60%	91.30%	
Sore throat	Yes	25.60%	31.50%	19.70%	0.063
	No	74.40%	68.50%	80.30%	
Headache	Yes	27.20%	28.30%	26%	0.118
	No	72.80%	71.70%	74%	
Cough	Yes	20.50%	24.40%	16.50%	0.068
	No	79.50%	75.60%	83.50%

**Table 3:** Ct value and melting temperatures (Tm) of the amplified serial dilution of the one step RT-qPCR and two step RT-qPCR

Standard dilution	Copies/ml	One step RT-qPCR RdRp gene	One-step RT-qPCR N gene	Two step RT-qPCR S gene		Two step RT-qPCR N gene	
		Ct (Threshold 2)	Ct (Threshold 2)	$\mathrm{Ct }(\mathrm{Threshold }0.2 )$	Tm (°C)	$\mathrm{Ct }(\mathrm{Threshold }0.2 )$	Tm (°C)
0.1	10^9^	19.97	16.44	18.56	83	19.93	85
0.01	10^8^	21.49	19.14	24.13	83	25.49	85
0.001	10^7^	24.03	21.56	27.52	83	28.14	85
0.0001	10^6^	27.5	23.82	32.06	83	32.31	85
0.00001	10^5^	31.56	27.31	36.05	83	36.91	85
0.000001	10^4^	34.66	30.77	39.41	83	39.18	85.14
0.0000001	10^3^	36.74	33.23	41.08	83.19	N.D	–
0.00000001	10^2^	40.01	37.83	N.D	–	N.D	–

### Analytical specificity of two-step RT-qPCR techniques

The analytical specificity results of the SYBR Green RT-qPCR showed no cross-attachment of the SARS-CoV-2 primers with OC43, 229E, NL63, H1N1, human rhinovirus, and human respiratory syncytial virus genes, indicating that the assay had well specificity.

### RT-qPCR techniques validation in clinical samples

**Fig. 2:**
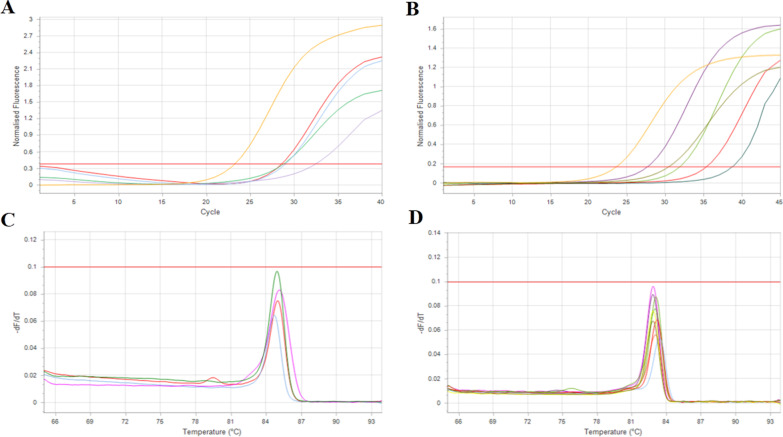
Two-step RT-qPCR amplification curves and amplicon of SARS-CoV-2-positive samples with one-step and two-step RTqPCR method and separation with 2% agarose gel electrophoresis. Amplification curves for positive samples using the N (**A**) and S (**B**) primer sets. The melt curve for positive samples using the N (**C**) and S (**D**) primer sets with specific peak

Each RT-qPCR manner was validated in terms of accuracy. We carried out the two-step and one-step RT-qPCR on positive and negative samples. The two-step method could detect 92% of the S gene and 88% of the N gene in positive samples. As well, it could recognize 84% of negative samples, correctly. Amplification and melt curves of clinical samples are shown in Fig. 2A–D. On the other hand, correctly, the one-step RT-qPCR method detected 92% of RdRp gene and 96% of N gene in positive and 86% of negative samples. The detection power of the one-step manner was 2.5% higher than that two-step method, but it was not significant (P = 0.086). The accuracy of the two-step method was 88% for the S gene and 86% for the N gene. The one-step was 86% for the RdRp gene and 94% for the N gene. In addition, the positive predictive value (PPV) and negative predictive value (NPV) in the two-step method for the S gene were 85% and 91%; also, they were 84% and 87% for the N gene, respectively. Furthermore, the one-step method had the PPV, and NPV values were 82% and 90% for the RdRp gene and 92% and 95% for the N gene, respectively (Table 4). There were no significant differences between PPV and NPV compared to the two methods (P > 0.05).

**Table 4:** Clinical performance of the compared one-step and two-step RT-qPCR for diagnosis SARS-CoV-2

Method	Target	Accuracy (%)	PPV (%)	NPV (%)
Two step RT-qPCR (SYBR Green-based)	S gene	88	85	91
	N gene	86	84	87
One step RT-qPCR (Prob-based)	RdRp gene	86	82	90
	N gene	94	92	95

### Visualization of RT-qPCR amplicon

**Fig. 3:**
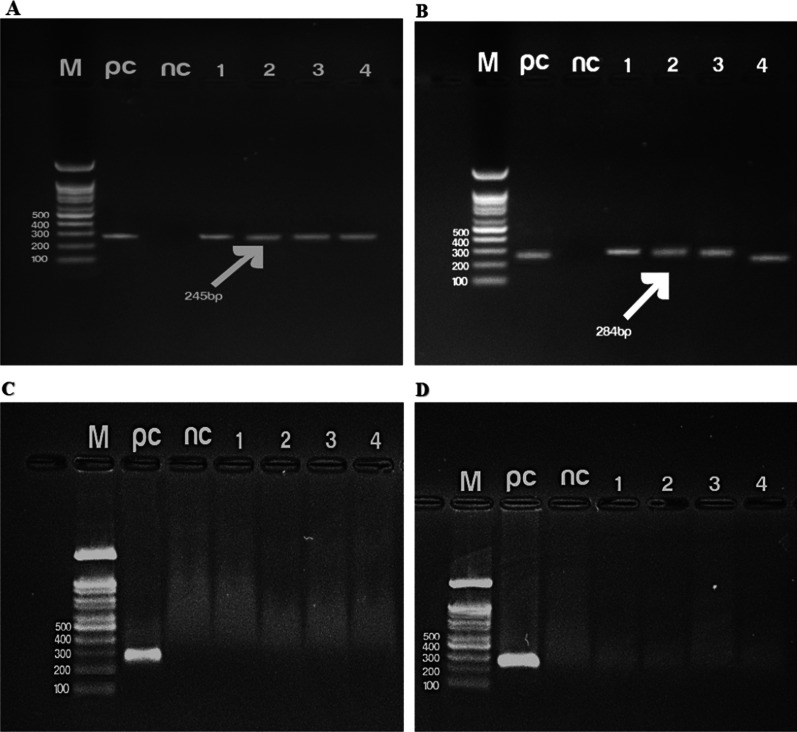
Visualization of RT-qPCR amplicon. Electrophoresis in agarose 1/5% gel of four positive clinical samples with positive and negative controls for the S (**A**) (245 bp) and N (284 bp) (**B**) primer sets. No band was created from the negative samples (**C**, **D**)

To investigate the specificity of amplification products, the two-step RT-qPCR amplicon of S and N genes were separated by gel electrophoresis. Positive samples of the S and N genes generated specific bands on the agarose gel (Fig. 3A and B). Then, the bands were visualized using a gel doc system. In addition, we did not observe any bands from negative samples (Fig. 3C and D).

### ROC curve analysis

**Fig. 4:**
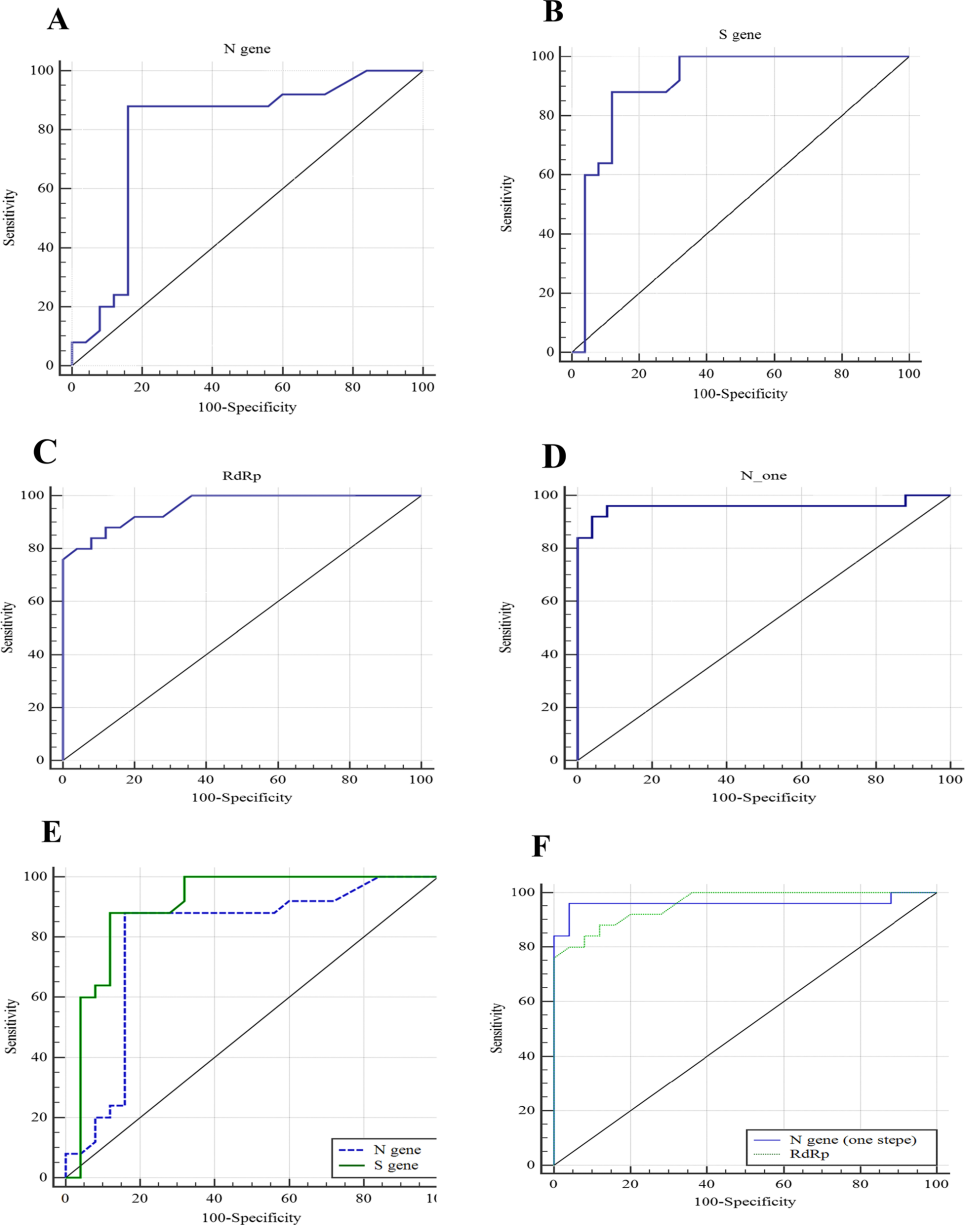
ROC curve analysis for detection of **A** N gene (one-step method), **B** RdRp gene (one-step method), **C** N gene (two-step method), **D** S gene (two-step method) **E** combination of two target S and N gene in two-step method, **F** combination of two target RdRp and N gene in one step method

To investigate and compare the potential diagnostic value of one-step and two-step RT-qPCR techniques to identify SARS-CoV-2, the ROC curve analysis was performed using MedCalc—version 14.8.1 (Fig. 4). The detection power of two-step method for N gene was revealed an area under the curve (AUC) of 0.79 (95% confidence interval (95% CI) 0.66 to 0.89; P = 0.048) with a sensitivity of 88% and specificity of 84% (Fig. 4A). Furthermore, the AUC of 0.90 (95% CI 0.79 to 0.97, P = 0.047) with a sensitivity 88% and specificity 88% were obtained for the S gene (Fig. 4B). The one-step manner showed a sensitivity of 92%, specificity of 80%, and the AUC of 0.95 (95% confidence interval (95% CI) 0.86 to 0.99, P = 0.023) for RdRp gene (Fig. 4C). Moreover, the AUC of 0.95 (95% confidence interval (95% CI) 0.86 to 0.99, P = 0.035) was calculated for N gene detection with a sensitivity of 92% and specificity 96% (Fig. 4D). The combination of the S gene and the N gene in two-step got the AUC 0.85 (95% CI 0.72 to 0.93) with 88% (68.8 to 97.5) sensitivity and 86% specificity (66.35 to 96.5) (P = 0.046) (Fig. 4E). Also, the combination of the RdRp gene and the N gene in one-step got the AUC 0.95 (95% CI 0.86 to 0.99) with 92% (74.45 to 99.8) sensitivity and 88% specificity (69.04 to 96.55) (P = 0.044) (Fig. 4F and Table 5).

**Table 5:** ROC curve analysis of two step RT-qPCR a and one-step RT-qPCR Ct

Target	Method	AUC (95% CI)	Sensitivity (%) (95% CI)	Specificity (%) (95% CI)	Cut-off value	P-value
S gene	Two step RT-qPCR	0.90 (0.79–0.97)	88 (68.8–97.5)	88 (68.8–97.5)	≤ 40	0.0471
N gene	Two step RT-qPCR	0.79 (0.66–0.89)	88 (68.8–97.5)	84 (63.9–95.5)	≤ 40	0.0489
RdRp gene	One step RT-qPCR	0.95 (0.86–0.99)	92 (74.0–99)	80 (59.3–93.2)	≤ 40	0.0234
N gene	One step RT-qPCR	0.95 (0.86–0.99)	92 (74.0–99)	96 (79.6–99.9)	≤ 40	0.0356
S and N gene	Two step RT-qPCR	0.85 (0.72–0.93)	88 (68.8–97.5)	86 (66.3–96.5)	≤ 40	0.0461
RdRp and N gene	One step RT-qPCR	0.95 (0.86–0.99)	92 (74.45–99.8)	88 (69.4–96.55)	≤ 40	0.0441

## Discussion

SARS-CoV-2 led to a global public health emergency state with the rapid spread and elevated morbidity and mortality rates [5]. Given the widespread epidemic of the SARS-CoV-2 virus and the possibility of a recurrence in the future, it is essential to provide an accurate, optimal, and low-cost diagnostic test for SARS-CoV-2 to prevent and control recurrence. The qPCR is a sensitive and specific method that does not need post-PCR steps. This assay is usually employed in medical diagnostic laboratories [20]. TaqMan probe-based RT-qPCR method is used to detect SARS-CoV-2 as a standard WHO protocol [3]. The Charity and Centers for Disease Control and Prevention (CDC) protocols use four different probes to determine a SARS-CoV-2 infection. These protocols contain reagents and probes that are infrequent and expensive [3]. The TaqMan probe-based RT-qPCR method is hard to access in the laboratories of developing countries [5]. In addition, frequent false results enhance the cost of the test as the sample measurement must be repeated 3-times [21].

The SYBR Green technique has a simple design and is a low-cost method but that is not specific. This method is mainly employed for the diagnosis and amplification of genomes. Generally, using specific primers leads to obtaining specific results [22]. Therefore, applying efficient primers in an SYBR Green-based method can turn into a low-cost alternative with high specificity and sensitivity [5]. The current study has evaluated the sensitivity and specificity of one- and two-step RT-qPCR for SARS-CoV-2 detection. PCR signals can be considerably impaired by the non-specific binding of primer [3]. Thus, we first assessed the specificity of the primers by the BLAST option on the NCBI website. In addition, we tested the positive clinical samples, including OC43, 229E, NL63, and Influenza a Virus (H1N1); human rhinovirus, a human respiratory syncytial virus with a two-step method in which it did not show any cross-reactivity.

In contrast to previous studies, our observations indicate that the SYBR green-based method is not precise for detecting SARS-CoV-2. Also, the sensitivity of the one-step method was higher than that two-step assay [22]. The results obtained from the diluted pool samples by the two-step RT-qPCR method detected amplification of the S and N genes up to 10^−6^. On the other hand, the one-step method detected all diluted pool samples’ concentrations. These results showed 100-fold more sensitivity for the one-step manner. It’s can be because of using the probes in this method [20]. More RNA or cDNA loadings can be added to the reaction mix to enhance the test’s sensitivity and improve the lower viral loads detection [5]. The mutations in target genes can also reduce the sensitivity of diagnostic tests and increase the false-negative results. Thus, monitoring the SARS-CoV-2 sequence for mutations that may affect the PCR assay is recommended [23]. The obtained results on positive clinical samples by the tow-step method showed a specific peak in the melting curve, revealing no non-specific products. Based on previous research, non-specific products during SYBR Green-based qPCR are considered false positives; accordingly, the melting curve should be analyzed [15]. In line with the previous investigation, our results showed that among assessed SARS-CoV-2 genes, the RdRp gene had the lowest specificity between the two-step and one-step techniques. This may be the conservation of the RdRp gene region among beta coronaviruses [24]. An occasional two-step analysis of negative samples may show unspecific sigmoidal fluorescence curves [14]. However, the stabile primers to targeted genes can significantly reduce such errors. Therefore, we propose that when RT-qPCR signal analysis is in linear mode, the negative result obtained from both primers can confirm the absence of the virus.

## Conclusion

The SARS-CoV-2 samples were recruited to evaluate the advantages and limitations of one-step and two-step qPCR methods. Our results proposed that the two-step method is practical for diagnosing SARS-CoV-2. We have set up two-step protocols that can be done using reverse transcriptase (MMLV-RT) and a variety of qPCR mixes. Combining this and easy RNA preparation methods would significantly improve the SARS-CoV-2 detection test capacities. Generally, the sensitivity and reliability of the two-step qPCR method are almost similar to the one-step qPCR manner. Besides decreasing expenses in medical diagnostic laboratories, this method will ease viral detection.

The obtained data from ROC curve analysis for every two manners of RT-qPCR showed specificity lower than 90%; however, we did not observe a cross-attachment of SARS-CoV-2 primers with other tested viruses. It’s maybe resulting from the reaction situation or the small sample size. More studies are needed to compare the one-step TaqMan probe-based method and the two-step SYBR Green-based method.

## Data Availability

All data generated or analyzed during this study are included in this published article.

## Abbreviations

COVID-19: Coronavirus disease 19
SARS-CoV-2: Severe acute respiratory syndrome coronavirus 2
RT-qPCR: Quantitative reverse transcription PCR
ddPCR: Droplet digital PCR
LAMP: Loop-mediated isothermal amplification
